# An Overview of Saturated Cyclic Ethers: Biological Profiles and Synthetic Strategies

**DOI:** 10.3390/molecules24203778

**Published:** 2019-10-21

**Authors:** Qili Lu, Dipesh S. Harmalkar, Yongseok Choi, Kyeong Lee

**Affiliations:** 1 College of Pharmacy, Dongguk University-Seoul, Goyang 10326, Korea; luqili220@gmail.com (Q.L.); dsharmalkar@gmail.com (D.S.H.); 2 College of Life Sciences and Biotechnology, Korea University, Seoul 02841, Korea; ychoi@korea.ac.kr

**Keywords:** saturated oxygen heterocycles, cyclic ethers, total synthesis

## Abstract

Saturated oxygen heterocycles are widely found in a broad array of natural products and other biologically active molecules. In medicinal chemistry, small and medium rings are also important synthetic intermediates since they can undergo ring-opening and -expansion reactions. These applications have driven numerous studies on the synthesis of oxygen-containing heterocycles and considerable effort has been devoted toward the development of methods for the construction of saturated oxygen heterocycles. This paper provides an overview of the biological roles and synthetic strategies of saturated cyclic ethers, covering some of the most studied and newly discovered related natural products in recent years. This paper also reports several promising and newly developed synthetic methods, emphasizing 3–7 membered rings.

## 1. Introduction

**Figure 1:**
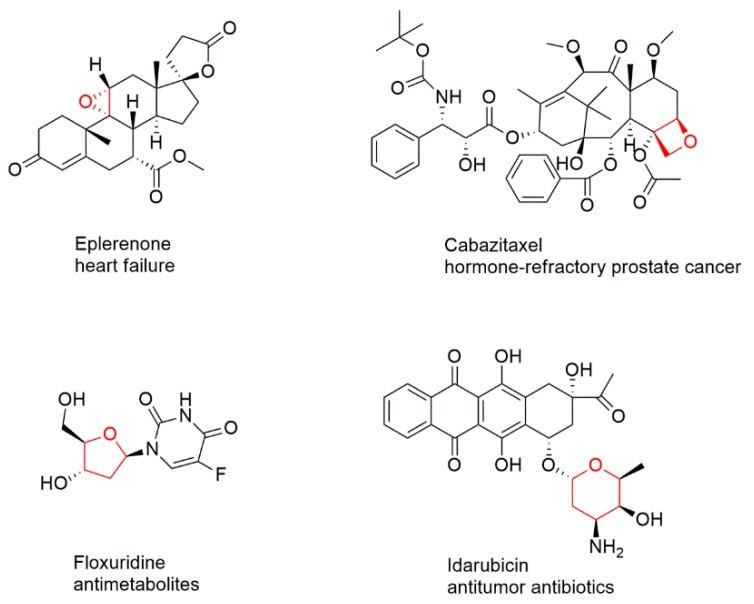
Structure and usage of FDA-approved drugs containing cyclic ether rings.

Constituting more than half of all the known organic compounds, heterocyclic compounds play an important role in organic chemistry. Among these, saturated cyclic ethers are abundant, appearing in a large number of biologically active natural products and pharmaceutically active compounds. The many FDA-approved cyclic ether rings containing therapeutic compounds (Figure 1) suggest and are evidence that cyclic ethers are significant motifs during the development of potential drug molecules. They have also been frequently found as key structural units in synthetic pharmaceuticals and agrochemicals. Additionally, a large number of natural products containing cyclic ethers have a wide range of interesting biological activities. For example, thousands of marine products that have oxacyclic moieties are isolated each year, providing rich sources for new drug candidates [1]. 

Over previous decades, considerable efforts have been devoted to the development of simple and efficient methods for constructing saturated oxygen heterocycles [2]. Given that most natural products occur as single enantiomers, and that chiral drugs on the market are regulated to be single enantiomers, special attention has been devoted to the asymmetric synthesis of heterocyclic compounds, as they play fundamental biological roles. 

Here, we attempted to provide an overview of saturated oxygen heterocycles. It would be an impossible endeavor to compose a comprehensive review of all the great achievements that have been made in this field. Therefore, we have collated the activities of some of the most heavily studied and newly discovered related natural products with the aim of showing the biological profiles of cyclic ethers. Several total synthesis methods are given as examples to show the general synthetic strategies used to generate cyclic ethers. We then glance at recent advances in the synthesis of cyclic ethers within the last 5 years that may be applied widely in organic synthesis in the future. 

## 2. Epoxides 

Epoxides are common in natural products and present a wide range of biological activities [3,4]. Synthetically, epoxides are very versatile intermediates [5]. Synthetic organic chemists can take advantage of regio- and stereoselective ring openings to easily convert epoxides into diols, amino alcohols, ethers, etc. [6]. Therefore, the formation of enantiomerically pure epoxides is an essential step in the asymmetric synthesis of organic chiral compounds. 

### 2.1. Natural Epoxides Containing Products and Biological Activities

**Figure 2:**
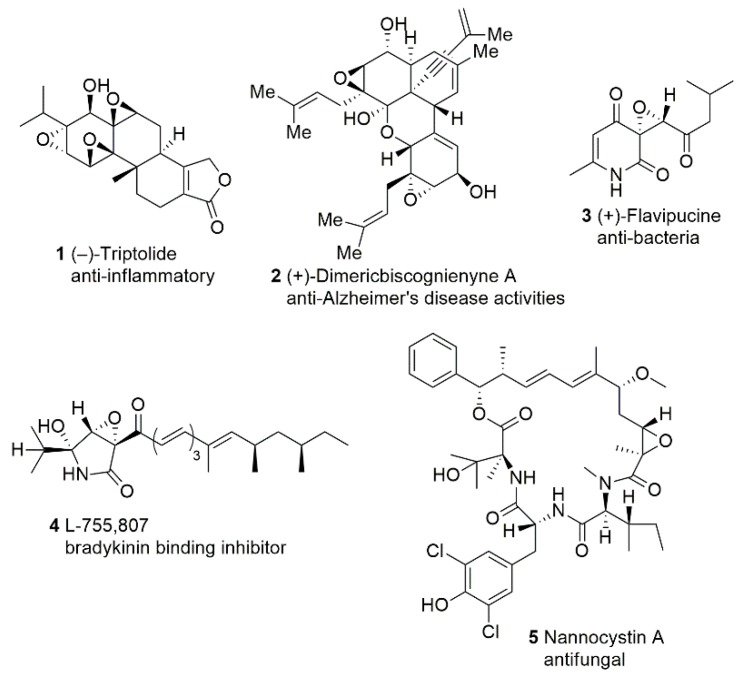
Epoxides containing natural products and biological activities.

Triptolide (TPL) **1** (Figure 2) is the major active component in an epoxy‑diterpene structure; it is isolated from *Tripterygium wilfordii* Hook. f. (TWHF), a vine-like plant widely distributed throughout Eastern and Southern China [7,8,9,10]. In Chinese traditional herbal medicine, the crude root extracts of TWHF have been used for centuries to treat autoimmune and inflammatory diseases such as rheumatoid arthritis and lupus erythematosus. TPL has also been recognized as a potential drug for a variety of cancers [11,12,13]. Recent research on TPL has been focused on mechanisms of action. Hu et al.’s studies showed that TPL significantly inhibited the growth of COC1/DDP cells (*p* < 0.05) at a low concentration of 3 ng/mL [14]. Animal results indicated that TPL + DDP significantly enhanced the inflammatory factor-2 (IL-2) and tumor necrosis factor-α (TNF-α) in serum of mice [15]. Song et al. [16] observed that TPL suppresses the growth of lung cancer cells by targeting hyaluronan-CD44/RHAMM signaling. Gao et al. [17] reported that TPL induces the proliferation and apoptosis of MCF‑7 breast cancer cells, potentially via autophagy and p38/Erk/mTOR phosphorylation. Minnelide, a more water-soluble synthetic analogue of TPL that is converted to TPL in vivo [18] has entered Phase II clinical trials for pancreatic cancer [19]. Triptolide is one of the most promising phytochemicals. 

In 2016, Zhao et al. [20] isolated five new compounds from secondary metabolites of *Biscogniauxia* sp., including the isolation of one new skeleton diisoprenyl-cyclohexene-type of meroterpenoid dimer—dimericbiscognienyne A **2** (Figure 2). In their anti-Alzheimer’s disease (AD) fly assay study, dimericbiscognienyne A showed short-term memory enhancement activities in AD flies [20]. 

(+)-Flavipucine **3** (Figure 2) is a pyridione epoxide isolated from the culture extract of *Phoma* sp., and Loesgen et al. [21] determined the absolute configuration by comparing the experimental and calculated CD spectra. Since its enantiomer (−)-flavipucine had been previously reported to possess antibacterial and antifungal activity [22,23], Loesgen et al. evaluated the biological activities of (+)-flavipucine **3** as well. To their delight, (+)-flavipucine **3** also exhibited good antibacterial and antifungal activity [21]. In 2019, Kusakabe et al. [24] synthesized and conducted antibacterial and cytotoxic evaluations of flavipucine and its derivatives. The antibacterial activity of the analogues, racemic flavipucine and both its enantiomers against Gram-positive *Bacillus subtilis* (*B. subtilis*) and Gram-negative *Escherichia coli* (*E. coli*), were evaluated via broth microdilution assay. Flavipucine was the most potent among the tested compounds. Furthermore, the results indicate that the pyridione epoxide moiety is a pharmacophore for antibacterial activity against *B. subtilis*. The cytotoxicity assay against cancer cells revealed that flavipucine has strong cytotoxic activity against HL-60 cells (IC_50_ = 1.8 µM). Surprisingly, there were no significant differences observed in the biological activity of the racemates or enantiomers of flavipucine [24]. 

Several natural products containing an epoxy-γ-lactam ring have been found to induce neurite outgrowth and are regarded as potential therapeutic agents for AD [25]. Tanaka et al. [26] devoted their interest to an epoxy-γ-lactam ring natural product, L-755, 807 (Figure 2). Isolated from an endophytic *Microsphueropsis* sp., L-755,807 consists of an epoxy-γ-lactam moiety and was identified as a bradykinin binding inhibitor with an IC_50_ of 71 μM. Tanaka et al. [27] completed the first total synthesis of (−)-L-755,807 and its stereoisomers. Recently, they carried out the establishment of relative and absolute configurations of L-755,807 and accomplished the structure−activity relationship (SAR) study for the first time. The biological evaluations revealed that the L-755,807 and its stereoisomers display potent inhibitory activities against amyloid-β aggregation (IC_50_ = 5–21 µM) which indicates that L-755,807 and related compounds could be a promising lead as compounds developed as therapeutic agents against AD [28]. 

Nannocystin A **5** (Figure 2), an epoxide-carrying compound isolated from a myxobacterium *Nannocystis* sp., was reported by Hoffmann et al. [29] and Krastel et al. [30] in 2015. According to Hoffmann et al., nannocystin A has a strong antifungal effect against *C. albicans* and displays potent cell proliferation inhibitive properties by inducing apoptosis early in tested cell lines. Parallel to Hoffmann’s research, Krastel et al. found that nannocystin A shows antiproliferative properties against 472 cancer cell lines in the nanomolar concentration range (IC_50_ values ranging from 0.5 μM to 5 nM). Moreover, combined genetic and proteomic approaches strongly suggest that the primary target protein of nannocystins is elongation factor 1-α (EF-1α). These studies indicate that nannocystin A may serve as a lead candidate for anticancer therapy. Due to the fact of its promising biological profiles, this novel 21 membered macrocycle immediately attracted chemists’ attention in 2016 [31,32,33,34]. Further SAR study by Tian’s group [35] demonstrated that the epoxide region does not interact directly with the bind site of the target eEF1a but is responsible for controlling the macrocyclic conformation.

### 2.2. Synthetic Strategies Used in Total Synthesis of Epoxides Containing Natural Products 

Oxidation of alkenes is a general strategy to provide epoxides. Peroxy acids, such as hydrogen peroxide (H_2_O_2_) and *meta*-chloroperoxybenzoic acid (mCPBA), are commonly used oxidizing agents [36,37,38,39]. 

**Scheme 1:**
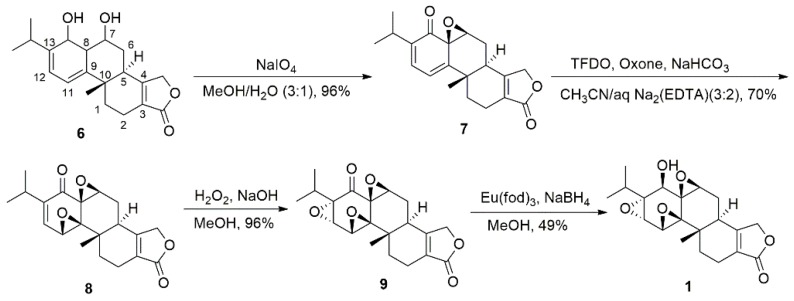
The generation of epoxide groups in the total synthesis of triptolide (TPL).

Yang et al. [40] completed the first enantioselective total synthesis of (−)-TPL in 2000. The fascinating structure and distinguished biological activity of TPL lead to considerable continued interest in their total synthesis and structure modification. In recent years, divergent total synthesis of TPL and its analogues have been reported [41,42,43,44]. However, these newly developed synthetic routes all adopted Yang et al.’s strategy to assemble the three successive epoxide groups (Scheme 1). Diol **6** was converted to monoepoxide **7** using the Adler reaction [45]. Epoxidation of **7** by in situ-generated methyl(trifluoromethyl)dioxirane (TFDO) and further epoxidation with alkaline hydrogen peroxide (H_2_O_2_/NaOH) successfully introduced the C9,C11 and C12,C13 epoxides, respectively, to give compound **9**. Reduction of **9** with NaBH_4_ in MeOH in the presence of Eu(fod)_3_ furnished (−)-TPL. 

The Darzens reaction is a non-oxidative method used to construct epoxycarbonyl from a halocarbonyl and an aldehyde in the presence of a base in organic solvents. The α- and β-Epoxy-γ-lactams are a highly valuable skeleton that can be generated from the Darzens reaction; they serve as attractive building blocks to access more complex molecular architectures. 

**Scheme 2:**
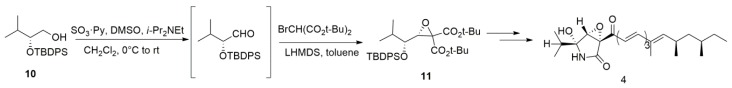
The total synthesis of L-755,807 using the highly diastereoselective Darzens reaction.

Tanaka et al. [27] accomplished the first asymmetric total synthesis of L-755,807 via a diastereoselective Darzens reaction (Scheme 2). Alcohol **10** was converted to an intermediate aldehyde using Parikh–Doering oxidation. Without further purification, the aldehyde was treated with bromo di-tert-butyl malonate pre-treated with lithium bis(trimethylsilyl)amide (LHMDS); the reaction proceeded cleanly to give only the desired diastereomer **11** in high yield. This strategy decreased the number of reaction steps and avoided side reactions. Further study showed that this reaction can be applied on aldehydes bearing a branched or an unbranched alkyl side chain. Moreover, two aromatic aldehydes were evaluated but a low yield was observed in each case [46]. The essential factor for high diastereoselectivity and yield might be attributed to the formation of a metal-cation-mediated rigid structure during the reaction [28]. Because researchers are still developing new chiral organocatalysts, the substitute scope of the highly enantioselective asymmetric Darzens reaction is expanding [47]. Moreover, shorter reaction times were achieved by using aqueous media in the presence of a Li^+^-containing base, a phase-transfer catalyst and granular polytetrafluoroethylene under mechanical stirring [48]. These developments make the Darzens reaction a promising method for the synthesis of natural products in an efficient and green way.

### 2.3. Recent Advances in Epoxidation

Due to the high need for enantiomerically pure epoxides, numerous powerful and efficient catalytic asymmetric reactions have been introduced and developed to generate epoxides [49]. Among these processes, Sharpless asymmetric epoxidation (SAE), Jecobsen–Katsuki epoxidation, Shi epoxidation, etc., are classic, powerful, and still popular [50,51,52]. The products of asymmetric epoxidation (AE) often show enantiomeric excesses above 90%. Nowadays, the challenge for AE is to explore more sustainable and efficient catalyst systems that are environmentally friendly. 

**Scheme 3:**
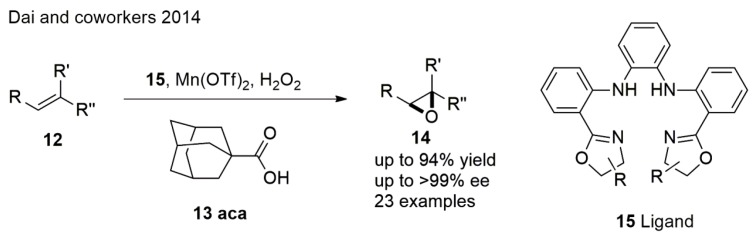
Synthesis of epoxides from trisubstituted olefins.

In an intriguing epoxidation of olefins with H_2_O_2_, Dai et al. [53] demonstrated that *cis, trans,* and terminal together with trisubstituted olefins can be converted to epoxides using an inexpensive and readily available in situ-formed manganese complex in excellent yields and enantioselectivities (Scheme 3). The additive adamantane carboxylic acid (aca) was found to be essential in improving the enantioselectivity. The supposed reason is that the sterically hindered aca could impart a highly rigid environment around the metal center.

**Scheme 4:**
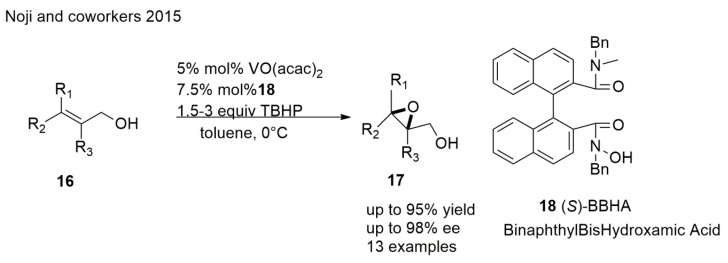
Synthesis of epoxides from allylic alcohols.

Sharpless asymmetric epoxidation of allylic alcohols is a reliable and commonly used method of obtaining chiral epoxy alcohols; it gives high asymmetric induction for various types of allylic alcohols and provides predictable configuration of the products. However, it requires strict anhydrous conditions. A good solution to this problem may be the development of vanadium–chiral hydroxamic acid (V−HA) complex-catalyzed AE. Noji et al. [54] disclosed a highly potent approach to obtaining chiral epoxy alcohols of 2,3,3-trisubstituted allylic alcohols using the vanadium–binaphthylbishydroxamic acid (BBHA) complex (Scheme 4). This method allows a simple reaction procedure which can be conducted in aqueous TBHP solutions and offers good yields and *ee*. Noji et al. [55] recently reported a great achievement in this field. They developed an immobilized polymer-supported vanadium-binaphthylbishydroxamic acid (PS-VBHA) that can be easily recycled and reused over five consecutive runs without significant sacrifice of catalytic activity or enantioselectivity. It would not be unrealistic to say that the application of chiral hydroxamic-acid ligands as enantioselective catalysts and the development of PS-VBHA will contribute to the application of sustainable green processes for various asymmetric oxidations.

**Scheme 5:**
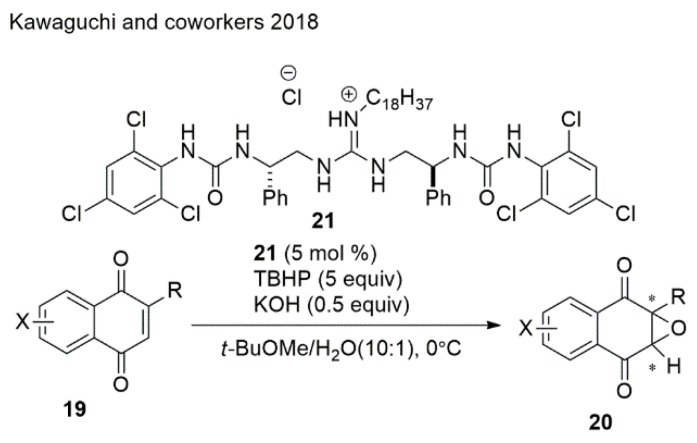
Synthesis of quinone epoxides from quinones.

Quinone epoxides are important synthetic intermediates for biologically active molecules. Nevertheless, it is difficult to apply AE on quinones because of their highly symmetric and planar structures with two carbonyl groups; therefore, differentiating the *si*- and *re*-faces of the olefins with chiral oxidants or catalysts is challenging. Kawaguchi et al. [56] developed an asymmetric epoxidation of 1,4-naphthoquinones catalyzed by guanidine−urea bifunctional organocatalysts with TBHP as an oxidant, resulting in the desired epoxides with 85:15−95:5 *er* in 71%−98% yields (Scheme 5).

## 3. Oxetanes 

Compared to epoxides, the oxetane ring appears in relatively few natural product structures, but when it is present, the natural products often display strong and intriguing biological activities. Oxetanes have been identified as efficient hydrogen-bond acceptors and have significant impacts on key physico- and biochemical properties [57]. Additionally, oxetanes are versatile building blocks of many other natural products and pharmaceutically active compounds [58]. Therefore, oxetanes have received a lot of interest as versatile precursors in synthetic chemistry [59,60].

### 3.1. Natural Oxetanes Containing Products and Biological Activities

**Figure 3:**
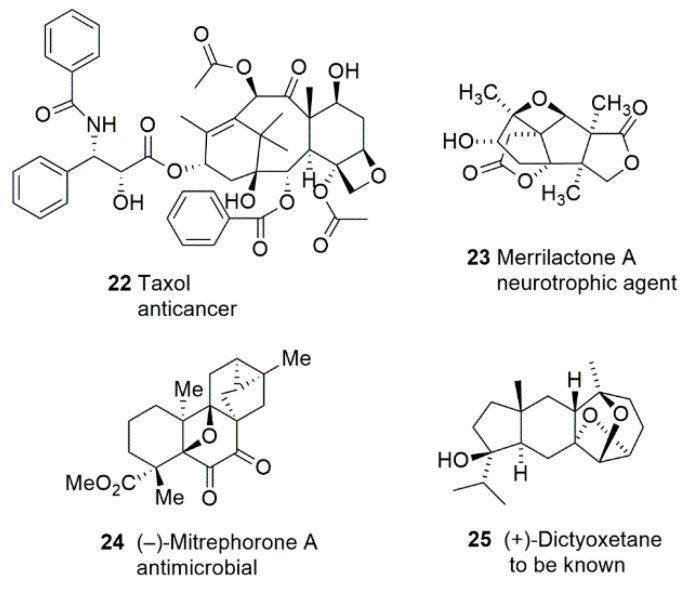
Oxetanes containing natural products and biological activities.

The most famous example could be Taxol (Figure 3) which is widely used to treat many types of cancers, such as breast cancer [61,62,63], ovarian cancer [64,65,66], lung cancer [67,68,69], cervical cancer [70,71,72], etc. Taxol and its semi-synthetic derivative cabazitaxel furthered interest in the study of oxetanes. Despite anticancer activity, oxetanes containing natural products exhibit many more pharmacological properties. Merrilactone A **23** has a unique sesquiterpene bearing two γ-lactones; an oxetane ring was isolated from the pericarps of *Illicium merrillianum*. The presence of an oxetane ring was required for neurotrophic activity. However, the isolation yield was only 0.004% [73]. Hence, total synthesis is fundamental for further biological research of merrilactone A. Mitrephorone A **24** is a compound isolated from the Bornean shrub *Mitrephora glabra* and displays potent cytotoxicity against a panel of cancer cells as well as featuring excellent antimicrobial activity [74]. It contains a fully substituted oxetane embedded in a pentacyclic carbon skeleton with a rare 1,2-diketone. This combination makes this natural product a veritable challenge for synthetic chemistry. The biological activities of some oxetanes containing natural products remain unavailable for many years due to the rareness and difficulty of chemical preparation, namely, (+)-dictyoxetane **25 [75]**. 

### 3.2. Synthesis Strategies Used in the Total Synthesis of Oxetanes Containing Natural Products 

Unlike epoxides, there is a paucity of synthetic methods available for the construction of oxetanes. In general, oxetanes can be synthesized via (a) Paternò–Büchi [2 + 2] photocycloaddition [76]; (b) C–O bond-forming cyclisation; (c) ring expansion of epoxides; and (d) C–C bond-forming cyclisation.

**Scheme 6:**
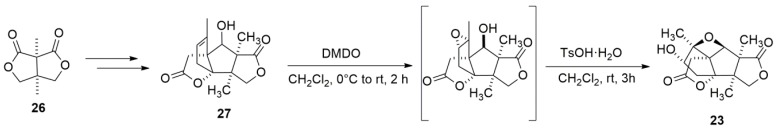
The total synthesis of merrilactone A.

A good example of using epoxide ring expansion to form oxetanes is the total synthesis of merrilactone A. Since its isolation in 2000, merrilactone A has been consistently appealing to researchers. Over the years, several different routes towards the total synthesis of merrilactone A have been reported [77,78,79,80,81]. Most recently, Liu and Wang [82] designed and achieved a concise synthesis of merrilactone A in a racemic form (Scheme 6). In their synthesis work, exploiting dimethyldioxirane (DMDO) on **27** allowed the epoxide intermediate that was subjected to acidic conditions to afford synthetic merrilactone A through the epoxide-opening oxetane formation. 

**Scheme 7:**
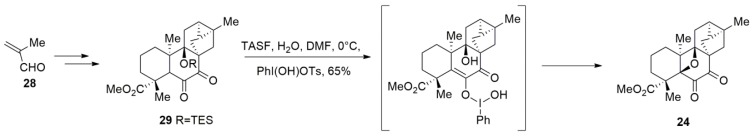
The total synthesis of mitrephorone A.

Richter et al. [83] reported the first and enantioselective synthesis of (−)-mitrephorone A in 2018 by using a novel late-stage oxidative cyclisation (Scheme 7). Synthesis commenced with methacrolein **28** and gave **29** in steps. Finally, the pivotal oxetane moiety was generated via one-pot deprotection of silyl ether **29** (TASF) and subsequent reaction with Koser’s reagent (PhI(OH)OTs) to successfully complete the total synthesis of (−)-mitrephorone A. 

### 3.3. Recent Advances in Oxetane Synthesis

**Scheme 8:**
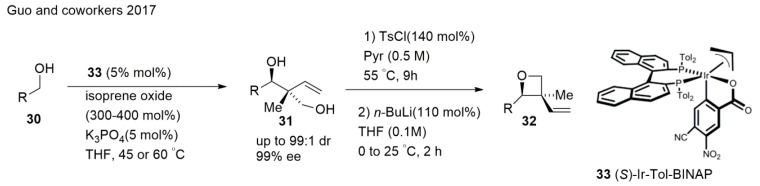
Synthesis of oxetanes using an iridium catalyst.

Impressive synthetic approaches toward C–C bond-forming hydrogenations and transfer hydrogenations were developed. Guo et al. [84] achieved access to oxetanes bearing all-carbon quaternary stereocenters readily prepared through the iridium catalyzed anti-diastereo and enantioselective C–C coupling of primary alcohols and isoprene oxide (Scheme 8). A group of primary alcohols **30** were exposed to isoprene oxide (300 mol%) and potassium phosphate (5 mol%) in the presence of the chromatographically purified π-allyliridium C,O-benzoate complex modified by (*S*)-Tol-BINAP in a tetrahydrofuran solvent at 60 °C to give adducts **31**. Conversion of the diol-containing adducts to the corresponding oxetanes **32** were accomplished through highly chemoselective tosylation of the primary alcohol moiety followed by SN_2_ cyclisation [84]. 

## 4. Tetrahydrofurans

Tetrahydrofuran (THF) moieties occur in many natural products with a wide array of bioactivities [85]; it has encouraged the development of a variety of synthetic methods [86,87].

### 4.1. Natural THF-Containing Products and Biological Activities

**Figure 4:**
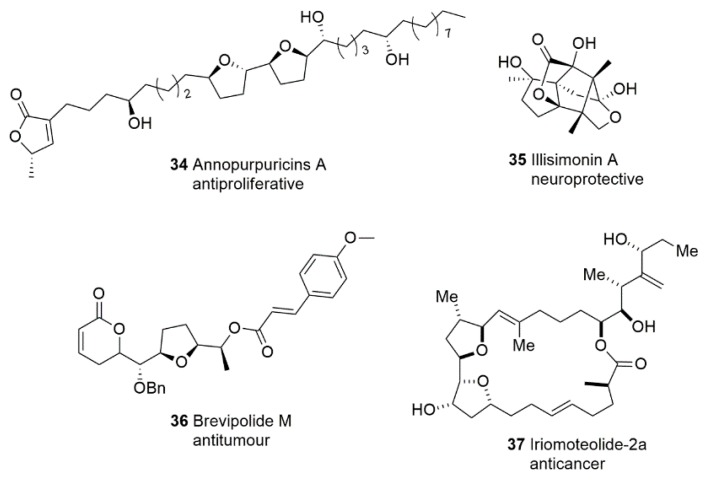
Tetrahydrofuran (THF)-containing natural products and biological activities.

Annonaceous acetogenins (AGEs) have been widely considered and extensively researched over the past three decades [88]. Structurally, AGEs are characterized by linear 32 or 34 carbon chains containing oxygenated functional groups. Most of them have one, two or three THF rings located along the hydrocarbon chain. Annonaceous acetogenins have been isolated from more than 50 different species of plants [89]. Extensive studies have indicated that members of this class of natural compounds possess a broad spectrum of bioactivity, featuring anticancer, antiparasitic, insecticidal, and immunosuppressive effects [90]. Recently, five new acetogenins were isolated from the roots of *Annona purpurea*, among which the most potent compound was annopurpuricins A **34** (Figure 4). Antiproliferative activity evaluation indicated that annopurpuricins A inhibited the growth of HeLa and HepG2 cells significantly with GI50 values of 0.06 nM and 0.45 mM, respectively. The THF rings may play an important role in these results [91]. The study of the antitumor mechanisms of acetogenins is also attractive to scientists [92].

The THF units are also found in many special skeletons. In 2017, Ma et al. [93] isolated illisimonin A **35** (Figure 4), which features a previously unreported tricyclic carbon framework from the fruits of *Illicium simonsii*. The structure and absolute configuration of illisimonin A were determined to be a caged 2-oxatricyclo[3,3,0,1^4,7^]nonane ring system fused to a five-membered carbocyclic ring and a five-membered lactone ring. These results were determined by the extensive use of spectroscopic evidence and electronic circular dichroism (ECD) calculations. Illisimonin A displayed potent neuroprotective effects against oxygen and glucose deprivation (OGD)-induced cell injury in SH-SY5Y cells, and its unique structure has inspired research into 3-Oxabicyclo [3.2.0]heptan-2-one core building blocks [94] and total synthesis [95].

In 2016, Suárez-Ortiz et al. [96] isolated and described the absolute configuration of five new compounds named brevipolides K−O from *H. brevipes*. Taking brevipolide M **36** (Figure 4) as an example, it contains a distinctive THF ring in the structure. These compounds displayed cytotoxicity against a variety of tumor cell lines including nasopharyngeal (KB) and cervix (HeLa) cancer cells with IC_50_ values of 1.7−10 μM.

Iriomoteolide-2a **37** (Figure 4) is a new anticancer macrolide isolated from the cultured broth of the benthic dinoflagellate *Amphidinium* sp. (HYA024 strain) collected off Iriomote Island, Okinawa, by the Tsuda group [97]. Significantly, it possesses potent cytotoxic activities against human B lymphoma DG75 cells and human cervix adenocarcinoma HeLa cells with IC_50_ values of 6 and 30 ng/mL, respectively.

### 4.2. Synthesis Strategies Used in Total Synthesis of THFs Containing Natural Products

Nucleophilic substitution chemistry plays an important role in the synthesis of cyclic ethers. Many THF-containing natural products have been constructed by employing intramolecular S_N_2 reactions between a hydroxyl group and a leaving group.

**Scheme 9:**
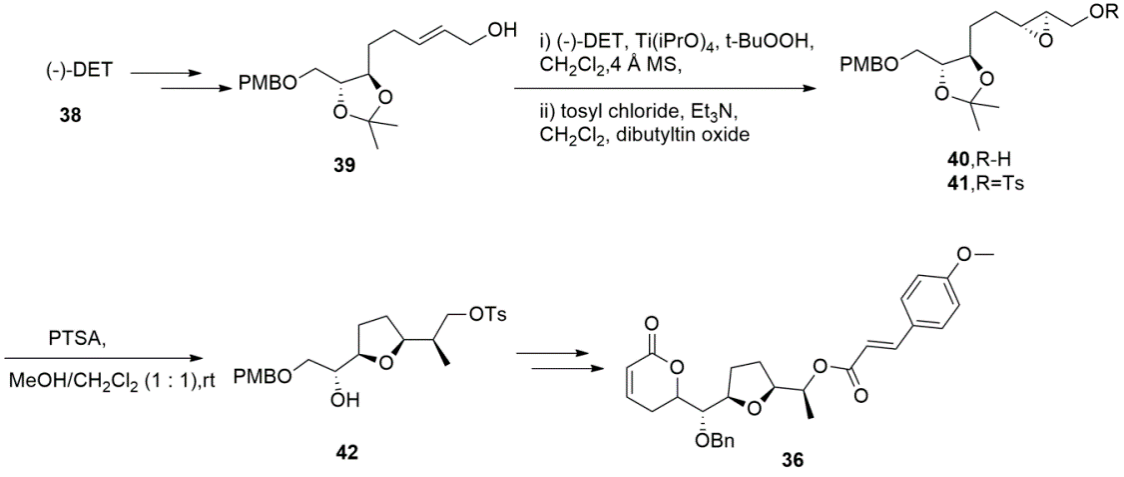
The total synthesis of brevipolide M.

Raju et al. [98] reported the first stereoselective total synthesis of brevipolide M with the readily available (−)-DET (Scheme 9). In this synthesis, allylic alcohol **39** was treated with Sharpless asymmetric epoxidation to provide the chiral epoxy alcohol **40** followed by tosylation of the primary alcohol. Treating tosyl compound **41** with PTSA in MeOH/CH_2_Cl_2_ (1:1) at rt for 2 h resulted in the deprotection of the acetonide group and the spontaneous cyclisation of hydroxy epoxide, giving the desired syn-tetrahydrofuran **42** in an 85% yield. Other key steps involved Brown’s allylation, the RCM reaction to install an α- and β-unsaturated lactone ring, and the inversion of the C-6′ stereogenic hydroxyl group using the Mitsunobu reaction furnished brevipolide M.

**Scheme 10:**
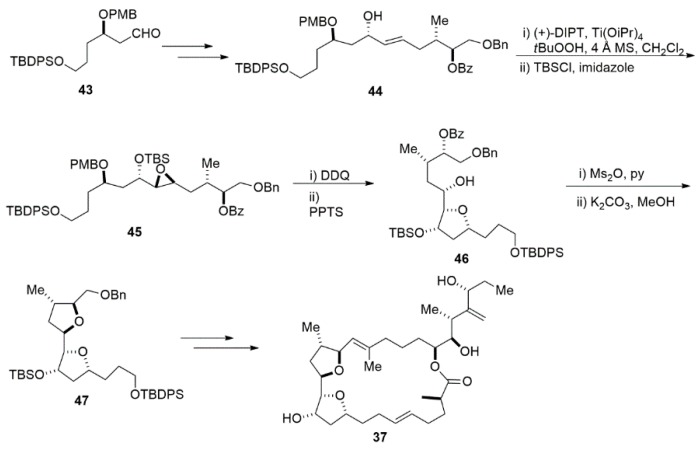
The total synthesis of iriomoteolide-2a.

Another example of THF construction is via the intramolecular addition of alcohols to epoxide. A good example of using this strategy is the total synthesis of iriomoteolide-2a by Sakamoto (Scheme 10) [99]. The gross structure of iriomotelide-2a consists of an unusual 23 membered macrocyclic backbone with a characteristic bis(tetrahydrofuran) substructure and a complex side chain containing four stereogenic centers. The efforts to achieve the bis(tetrahydrofuran) unit is a fundamental part of total synthesis. In Sakamoto’s approach, Sharpless epoxidation of **44** followed by the first THF ring formation using the intramolecular addition of alcohols to epoxide gave alcohol **46**. After mesylation, cleavage of the benzoyl group and concomitant cycloetherification in the basic condition furnished bis(tetrahydrofuran) **47** [100].

### 4.3. Recent Advances in THF Synthesis

**Scheme 11:**
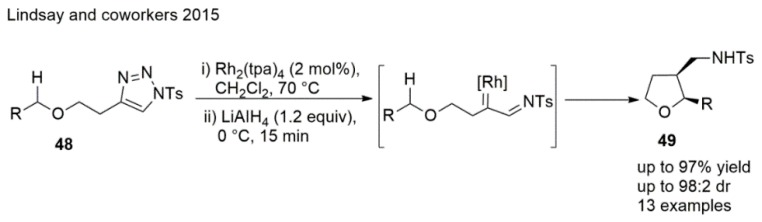
Synthesis of THF using C(sp3)−H insertion.

A stereodivergent intramolecular Rh-catalyzed azavinyl carbenoid C(sp^3^)−H insertion reaction was achieved by Lindsay et al. (Scheme 11), which allowed the formation of *cis*-2,3-disubstituted THFs. Good yields were observed and the resulting THF products were transformed to ring-fused THFs efficiently [101].

**Scheme 12:**
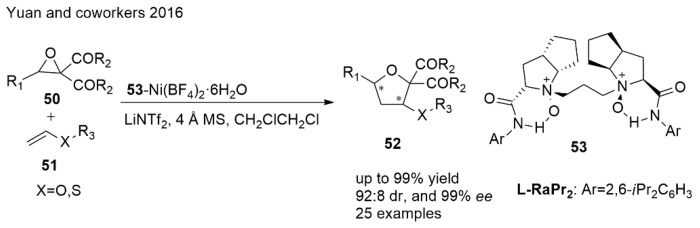
Synthesis of THF using [3 + 2] cycloaddition.

Oxiranes have become interesting precursors in the last few years. Yuan et al. [102] demonstrated an efficient diastereo- and enantioselective [3 + 2] cycloaddition of heterosubstituted alkenes with oxiranes via selective C−C bond cleavage of epoxides to give chiral THFs (Scheme 12). The reaction was catalyzed by a chiral *N,N*′-dioxide/Ni(II) catalyst which was derived from L-ramipril (Ra) by complexing with Ni(BF_4_)_2_·6H_2_O. The enantioselectivity was found to increase little by little as the steric hindrance at the *ortho* positions of the aniline of *N,N*′-dioxide ligands or on the heterosubstituted alkenes became larger.

**Scheme 13:**
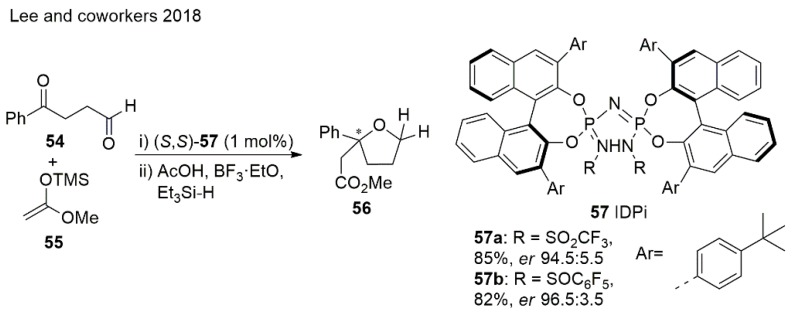
Synthesis of THF using an IDPi catalyst.

Asymmetric nucleophilic additions to keto aldehydes in the presence of enantiomerically pure imidophosporimidates (IDPis) interestingly shows that ketone reacts preferentially over the aldehyde. This method provides 2,2-disubstituted THF analogues with tetrasubstituted stereogenic centers starting from 1,4-dicarbonyl compounds [103] (Scheme 13). Moreover, 2,2,5,5-tetrasubstituted THFs can be readily prepared using the described method.

## 5. Tetrahydropyrans

Tetrahydropyrans (THPs) have received a considerable amount of attention from many biologists and synthetic organic chemists due to the prevalence of these substructures in biologically interesting natural products [104,105].

### 5.1. Natural THPs Containing Products and Biological Activities

**Figure 5:**
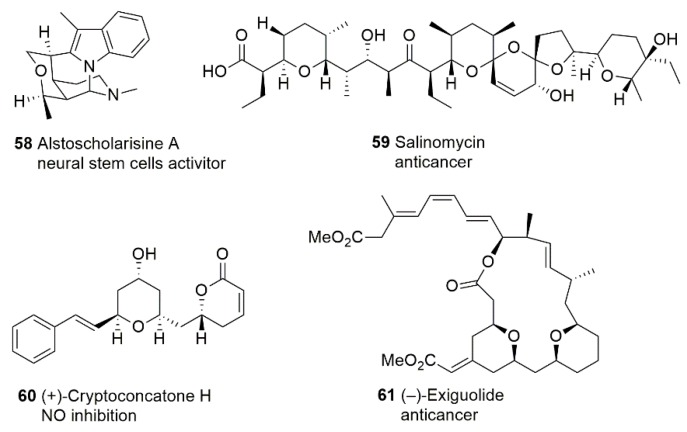
THPs containing natural products and biological activities.

Salinomycin (SAL) **59** (Figure 5) has shown a broad spectrum of bioactivity, including antibacterial, antifungal, antiviral, antiparasitic, and anticancer activity, proving its significant therapeutic potential [106,107]. Many research groups around the world are currently performing intensive studies to discover novel aspects of the biological activity of SAL and its derivatives. Namely, Tyagi et al. [108] recently reported that a follow-up treatment of SAL may be a promising strategy against cisplatin (*cis-*diamminedichloro-platinum, CDDP)-resistant breast cancer cells and metastasis and help reduce CDDP-induced side effects as it reduces the growth, proliferation, and metastasis of cisplatin-resistant breast cancer cells via NF-kB deregulation. Another discovery of SAL’s effects on breast cancer by Dewangan et al. indicated that SAL inhibits breast cancer progression via targeting HIF-1α/VEGF-mediated tumor angiogenesis [109].

In 2014, Yang et al. [110] isolated five unprecedented monoterpenoid indole alkaloids from *Alstonia scholaris*. The most potent compound, alstoscholarisine A **58** (Figure 5), promoted adult neural stem cell proliferation significantly with a concentration of 0.1 μg/mL in a dosage-dependent manner and did not affect the proliferation of neuroblastoma cells. This finding attracted interest from the synthetic community to achieve the total synthesis of this series of compounds with a new skeleton [111]. In 2016, Yang et al. [112] published the new compounds they isolated from the leaves and twigs of *C. concinna*. Among them, the structure of cryptoconcatone H **60** (Figure 5) was proposed as an *S* absolute configuration through the interplay of Mosher’s ester methodology and ROESY experiments whereas Della-Felice et al. revised it as all *R* stereoisomer shown in Figure 5 [113]. Cryptoconcatone H displayed the inhibition of NO production induced by LPS in RAW 264.7 macrophages with an IC_50_ value of 4.2 µM [112].

### 5.2. Synthesis Strategies Used in the Total Synthesis of THP-Containing Natural Products

A large number of valuable and high-quality contributions have been made in the construction of THP rings [104,114]. The Prins cyclisation reaction and its variants are extremely powerful methods for constructing THF [115]/THP [116] rings and widely applied in total synthesis [117,118,119].

**Scheme 14:**
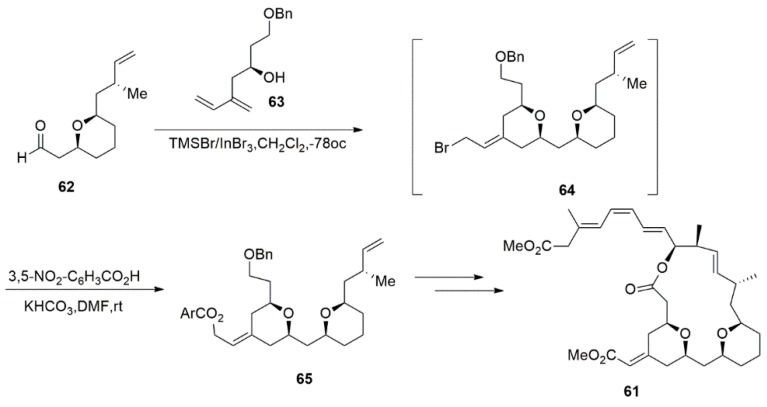
Total synthesis of (−)-exiguolide.

Li et al. [120] described a Prins cyclisation/homobromination process that involved dienyl alcohol with aldehyde to construct *cis*-THP containing an exocyclic *E*-alkene by using TMSBr/InBr_3_ as a combined bromide source and a Lewis acid. This approach provides good-to-excellent *cis*-E stereochemical control in one step, and this reaction was soon employed in the total synthesis of (−)-exiguolide **61** (Scheme 14). The ester substituted **65** was delivered at 71% yield with a *cis/trans* ratio of ≥95:5 and a *Z/E* ratio of 95:5 [121].

**Scheme 15:**
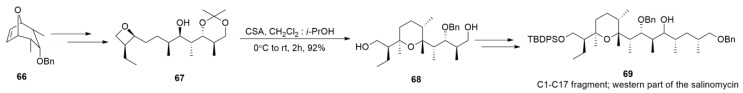
Synthesis of the C1–C17 fragment of salinomycin.

The THP formation was observed during Jacobsen et al.’s study of the enantioselective intramolecular opening of oxetanes for obtaining enantioenriched THFs [122]. In 2014, Yadav et al. [123] disclosed that exposing oxetanes to acids in the presence of aprotic solvents can afford THPs smoothly. The best solvent condition was found to be a mixture of CH_2_Cl_2_ and *i*-PrOH (15:1), in which oxetane substrates were converted into THPs at high yields within two hours. This newly developed methodology was successfully applied to the synthesis of the THP motif of salinomycin (Scheme 15).

### 5.3. Recent Advances in THP Synthesis

**Scheme 16:**
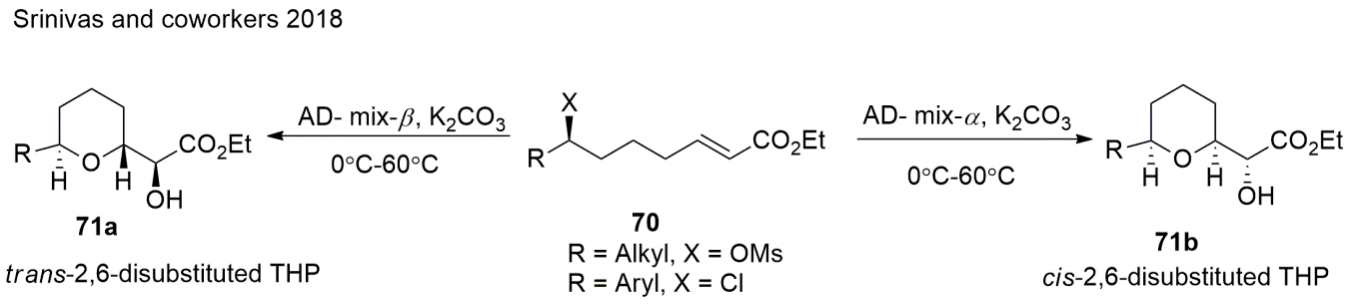
Synthesis of both *cis*- and *trans*-THPs from a common precursor.

Srinivas et al. [124] demonstrated an efficient one-pot protocol to obtain THPs via the diastereoselective tandem dihydroxylation of ζ-mesyloxy α,β-unsaturated esters followed by SN_2_ cyclisation (Scheme 16). The highlight of this method is that it allows both *cis*- and *trans*-THP rings to be synthesized starting from a common precursor. This protocol also demonstrated the formal synthesis of (+)-muconin in a concise and highly stereoselective manner.

**Scheme 17:**
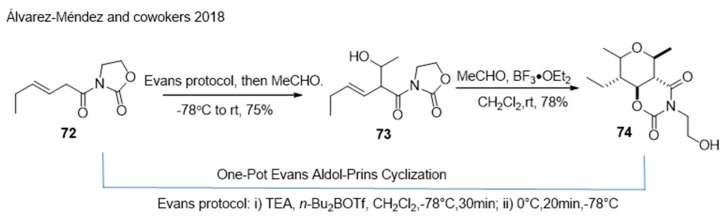
One-Pot Evans–Prins cyclisation to construct THPs.

In 2018, Sergio [125] reported a direct and general method for the synthesis of naturally occurring 2,3,4,5,6-pentasubstituted THPs employing β,γ-unsaturated N-acyl oxazolidin-2-ones as key starting materials (Scheme 17). The combination of the Evans aldol addition and Prins cyclisation allowed the diastereoselective and efficient generation of the target highly substituted THPs.

## 6. Oxepanes

**Figure 6:**
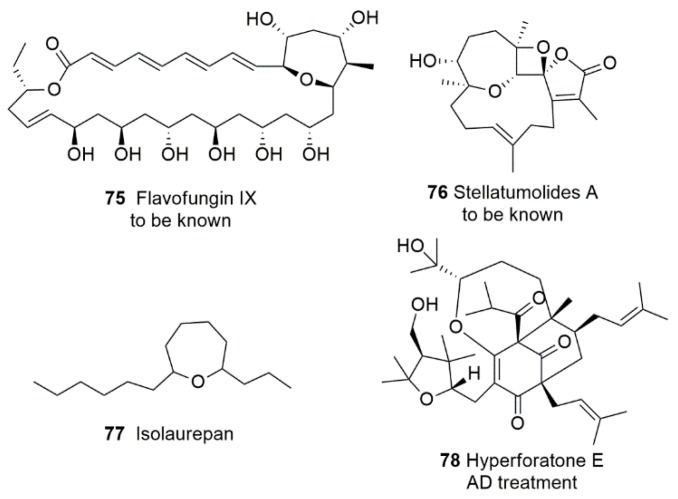
Oxepane-containing natural products and biological activities.

Natural products containing oxepane scaffolds are not rare, particularly among marine products. Their interesting biological properties can include anticancer, antibacterial, and antifungal activities. Oxepane motif in known natural products examples is often flanked by aromatic moieties or polycyclics [126,127], making the structures overwhelmingly complex and unusual. Simple oxepane compounds, such as isolaurepinnacin **77** (Figure 6), have been synthetic targets of considerable interest.

### 6.1. Natural Oxepane-Containing Products and Biological Activities

Only a few new oxepane-containing natural products have been isolated in recent years. In 2017, flavofungin IX **75** (Figure 6) was obtained by Wang et al. from mangrove-derived *streptomyces* sp. ZQ4BG [128]. In 2018, Ahmed et al. isolated stellatumolides A **76** (Figure 6) from soft coral *Sarcophyton stellatum* [129]. However, there have been no further reports of these new compounds. It would be interesting to discover their bioactivities or synthetic methods. Guo et al. isolated 15 new polycyclic polyprenylated acylphloroglucinols (PPAPs) from the stems and leaves of *Hypericum perforatum*, including six oxepane ring-containing products hyperforatones I-J [130]. Hyperforatone E **78** (Figure 6) was found to exhibit dual inhibitory activities against AChE and BACE1. Preliminary molecular docking studies have shown that it has strong interactions with the major active sites of BACE1 and AChE. These initial studies suggest that hyperforatone E may be further developed into a potential candidate or lead compound for AD treatment.

### 6.2. Synthesis Strategies Used in the Total Synthesis of Oxepane-Containing Natural Products

The oxepanyl rings in natural products are commonly bonded to a great diversity of rings and chains. Very few total syntheses of such complicated oxepane-containing compounds have been achieved in recent years due to the challenge of oxepane formation. The development of the construction of oxepane motifs would be beneficial for the synthesis of bioactive molecules and the total synthesis in the future.

**Scheme 18:**
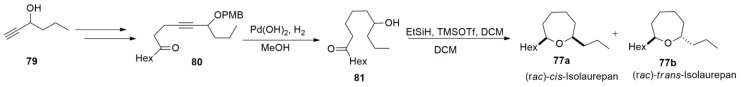
Synthesis of isolaurepan.

In 2016, Hernàndez-Torres et al. [131] developed a synthesis method of 7–9 membered cyclic ethers by the reductive cyclisation of hydroxy ketones and successfully applied this to the total synthesis of isolaurepan (Scheme 18). Full hydrogenation and PMB cleavage of **80** under hydrogen atmosphere afforded hydroxy ketone **81**. Then, reductive cyclisation by treatment with Et_3_SiH and TMSOTf in CH_2_Cl_2_ gave the desired products. Even though many synthesis routes towards isolaurepan have been reported, this methodology stands out for its few steps and comparably high yield.

### 6.3. Recent Advances in Oxepane Synthesis

**Scheme 19:**
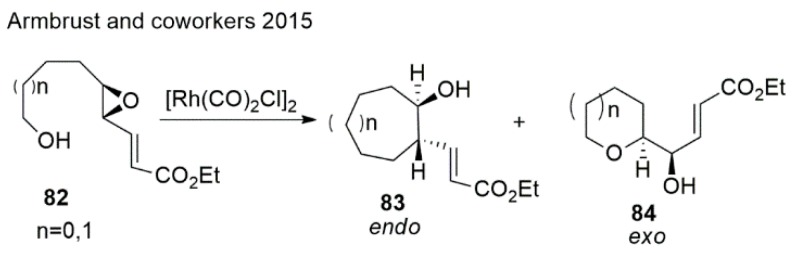
Synthesis of oxepanes by cascade reaction.

Armbrust et al. [132] disclosed a successful approach to assembling 6 and 7 membered oxygen heterocycles (Scheme 19). In their method, di/trisubstituted epoxides were activated by Rhodium catalyzed via π-coordination and oxidative addition into the vinylic C−O bond of the epoxide.

**Scheme 20:**
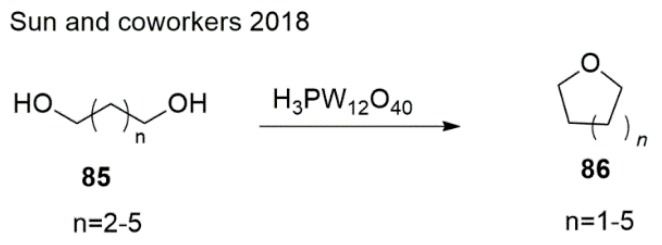
Synthesis of oxepanes by HPA-catalyzed cyclodehydration.

The cyclodehydration of diols offers good access to 7 membered rings. In 2018, Sun et al. [133] achieved oxepane via the heteropoly acids (HPAs)-catalyzed cyclodehydration of hexane-1,6-diol in 80% yield (Scheme 20). There are no substituted oxepane examples given in the literature. It would be interesting to try making substituted oxepanes using this protocol.

## 7. Conclusions

This review, although covering only a small fraction of cyclic ether chemistry, demonstrates that small- and medium-ether rings appear in a large variety of natural resources and most of these compounds display promising biological and pharmacological properties, such as antibiotic, antibacterial, and antitumor activities. Even though cyclic ethers are not privileged heterocycles, they represent an undeniably important class of compounds. Many reliable synthetic methods have been efficiently applied to the total synthesis of related natural products, while numerous new developments of cycle ether synthesis have been achieved in organic chemistry. However, there is still a large developing space left to explore: discovering and creating new pharmacologically active molecules, extending the substitute scope of synthetic strategies, overcoming the difficulty of constructing large rings, and achieving new drugs.
